# Growth hormone increase by luteinizing hormone-releasing hormone reflects gonadotroph-related characteristics in acromegaly

**DOI:** 10.1007/s11102-024-01410-2

**Published:** 2024-06-19

**Authors:** Yuto Mitsui, Kosuke Mukai, Michio Otsuki, Satoru Oshino, Youichi Saitoh, Masaharu Kohara, Eiichi Morii, Atsunori Fukuhara, Iichiro Shimomura

**Affiliations:** 1 Department of Metabolic Medicine, Osaka University Graduate School of Medicine, 2-2 Yamada-oka, Suita, Osaka 565-0871 Japan; 2 Department of Endocrinology, Tokyo Women’s Medical University, 8-1 Kawada-cho, Shinjuku-ku, Tokyo, 162-8666, Japan; 3 Department of Neurosurgery, Osaka University Graduate School of Medicine, Osaka, Japan; 4 Tokuyukai Rehabilitation Clinic, Osaka, Japan; 5 Department of Pathology, Osaka University Graduate School of Medicine, Osaka, Japan; 6 Department of Adipose Management, Osaka University Graduate School of Medicine, Osaka, Japan

**Keywords:** Acromegaly, LHRH, Somatostatin, Steroidogenic factor 1, Gonadotropin

## Abstract

**Purpose:**

We previously showed the clinical characteristics of acromegaly with a paradoxical growth hormone (GH) response to oral glucose or thyrotropin-releasing hormone. However, the clinical characteristics of acromegaly with an increased GH response to luteinizing hormone-releasing hormone (LHRH responders) remain unclear. The aim of the present study was to evaluate the clinical characteristics, especially gonadotroph-related characteristics of LHRH responders in acromegaly.

**Methods:**

The clinical characteristics of 33 LHRH responders and 81 LHRH nonresponders were compared.

**Results:**

No differences in age, sex or basal serum levels of GH, insulin-like growth factor-1 (IGF-1), and gonadotropin were observed between the two groups. Steroidogenic factor 1 (SF-1), gonadotropin-releasing hormone receptor (GnRHR), and LH expression was more frequently observed in LHRH responders (*P* < 0.05). In addition, a greater increased rate of GH after LHRH loading, and the proportion of GnRHR and gonadotropin expression was observed in pituitary tumor with SF-1 expression than that without the expression (*P* < 0.01). LHRH responders showed a greater GH decrease in the octreotide test and a greater IGF-1 decrease after first-generation somatostatin ligand than LHRH nonresponders (*P* < 0.05). Furthermore, the proportion of hypointense pituitary tumors on T2-weighted magnetic resonance imaging and tumors with densely granulated type was higher in LHRH responders than in LHRH nonresponders, respectively (*P* < 0.05). No difference between the two groups was observed in either somatostatin receptor 2 or 5 expression.

**Conclusions:**

The increased GH response to LHRH is associated with the gonadotroph-related characteristics. This response may reflect the biological characteristics of somatotroph tumors.

## Introduction

We previously reported the clinical characteristics including the efficacy of first-generation somatostatin ligand (fg-SRL) in acromegaly patients with an increased growth hormone (GH) response to the oral glucose tolerance test (OGTT) or thyrotropin-releasing hormone (TRH) loading [1, 2]. Furthermore, other groups showed similar results [3, 4]. In addition to OGTT and TRH loading, luteinizing hormone-releasing hormone (LHRH) loading induces an increased GH response in some acromegaly patients (LHRH responders) [5]. However, no study has examined the clinical characteristics of acromegaly patients with an increased GH response to LHRH loading.

LHRH induces the secretion of LH and follicle stimulating hormone (FSH) from gonadotrophs in the normal pituitary gland. Pituitary tumors in LHRH responders of acromegaly may have the phenotype of gonadotroph in addition to that of somatotroph because LHRH can stimulate GH secretion from a pituitary tumor in LHRH responders as well as the secretion of LH and FSH from gonadotroph. Therefore, pituitary tumors in LHRH responders of acromegaly might express steroidogenic factor 1 (SF-1), a gonadotroph transcription factor, as the phenotype of gonadotroph in addition to pituitary-specific positive transcription factor 1 (PIT-1) as that of somatotroph. Moreover, pituitary tumors of LHRH responders in acromegaly might express gonadotropin-releasing hormone receptor (GnRHR) and gonadotropin. The relationship between the expression of pituitary transcription factors and the clinical characteristics of acromegaly has not been elucidated although the World Health Organization (WHO) reclassifies pituitary tumors according to their pituitary transcription factors in 2022 [6].

In the present study, we investigated the clinical and pathological characteristics, including SF-1, GnRHR, LH, and FSH expression, and efficacy predictors of medical treatment of LHRH responders.

## Subjects and methods

### Subjects

A retrospective analysis was conducted on 114 patients with acromegaly (112 untreated patients and 2 postoperative patients with active acromegaly) who were hospitalized at Osaka University Hospital from April 2004 to January 2020, including patients from a previous study [1]. The diagnosis of acromegaly was determined based on clinical features, serum GH levels that remained unsuppressed to < 0.4 ng/ml in the 75-g OGTT, insulin-like growth factor-1 (IGF-I) levels that exceeded the upper limit of normal, and the identification of a pituitary tumor on magnetic resonance imaging (MRI). The study population consisted of 60 women and 54 men with a median age of 54 years [interquartile range (IQR), 42 to 60 years] and a body mass index of 24.2 kg/m^2^ (IQR 22.1 to 26.8). The human ethics committee of Osaka University approved the study (approval no. 16136), which was conducted in accordance with the Declaration of Helsinki.

### Endocrinological evaluation

**Fig. 1:**
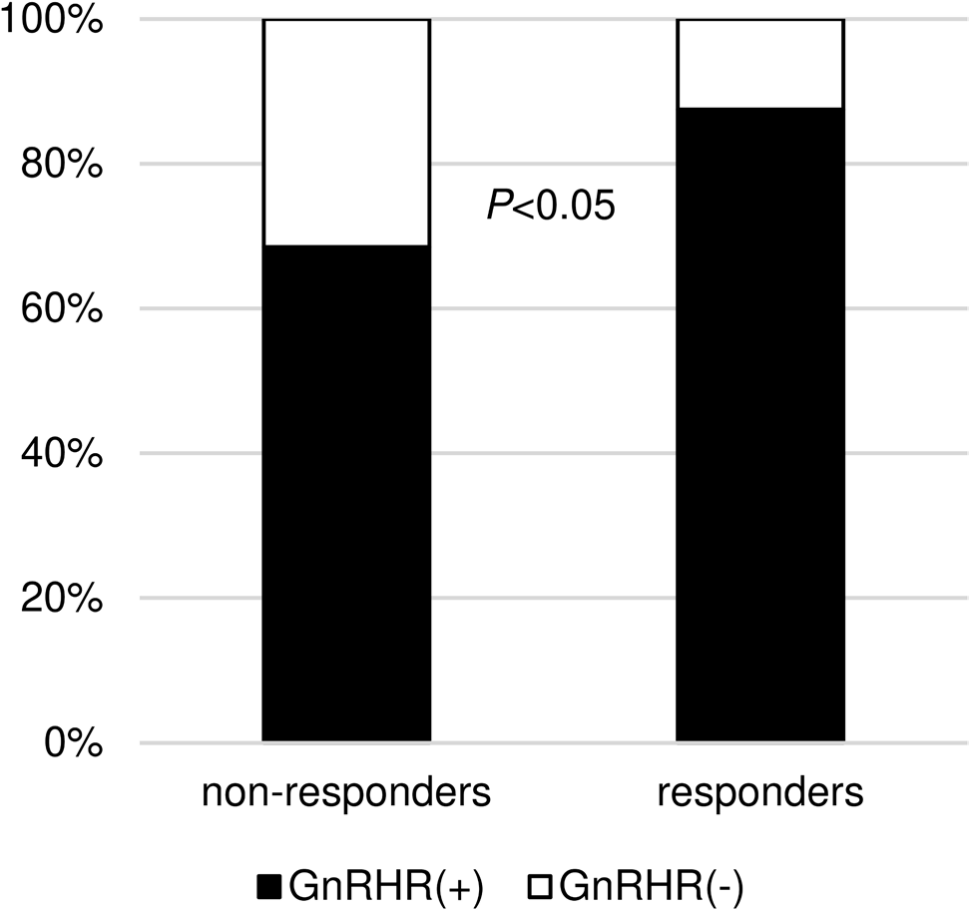
Histopathological classification by GnRHR expression, in LHRH nonresponders and responders

Fasting basal serum GH and IGF-I, LH, and FSH levels were measured without acromegaly medical treatment including fg-SRL and pituitary surgery. GH was measured before and 30, 60, 90, and 120 min after 0.1 mg of LHRH loading. It was reported that LHRH had less impact on GH secretion in LHRH nonresponders [5]. An increased GH response to 0.1 mg of LHRH loading was defined as a 30% increase in the peak serum GH level from the basal GH level after LHRH loading because there was a significant difference of GnRHR expression in pituitary tumors between LHRH responders with ≥ 30% of GH increase and LHRH nonresponders (Fig. 1). A paradoxical GH response to the 75-g OGTT was defined as a 20% or 30% increase in the serum GH level from the basal GH level after OG loading [1, 3, 7]. Similarly, an increase GH response to 0.5 mg of TRH loading was defined as a 100% increase in the serum GH level [2]. The response to 2.5 mg of oral bromocriptine or 50 µg of subcutaneous octreotide was also evaluated and expressed as a percentage: (nadir GH level − basal GH level)/basal GH level. The serum GH levels were measured before and 1, 2, 4, 6, 8, 12, and 24 h after bromocriptine or octreotide administration. The OGTT and bromocriptine and octreotide tests were performed in 111, 111, and 112 patients, respectively. The serum GH level was measured using an immunoradiometric assay kit (GH kit Daiichi; Daiichi Radioisotope Laboratories, Tokyo, Japan) until April 2007, by a chemiluminescent enzyme immunoassay kit (Access hGH kit; Beckman Coulter, Tokyo, Japan) until April 2017, and by an Electro Chemiluminescence Immunoassay kit (ECLusys hGH; Roche Diagnostics, Tokyo, Japan) until the end of the study. The measured values were adjusted using a correlation formula estimated by a linear regression model. The GH levels measured by the first and second kits were converted to values consistent with the third kit using the following formula: y = 1.4x (y: value used for judgment, x: measured value by the first and second kits). The GH levels using the third kits were directly employed without necessitating any form of adjustment. The IGF-I level was measured using an immunoradiometric assay kit (Daiichi Radioisotope Laboratories) until October 2019 and an Electro Chemiluminescence Immunoassay kit (ECLusys IGF-1; Roche Diagnostics, Tokyo, Japan) until the end of the study. The standard deviation score of IGF-1 (IGF-1 SD score) was calculated using the standardized centile curves and reference intervals of IGF-1 levels in a healthy Japanese population [8]. Three of seven patients without pituitary surgery were treated by only fg-SRL. On the other hand, preoperative fg-SRL treatment for 31 patients were performed. Additionally, 54 (50%) and 27 (25%) of 107 patients with pituitary surgery for acromegaly at our hospital could achieve remission (both nadir GH after OGTT < 0.4 ng/ml and normal IGF-1) and control (both nadir GH after OGTT ≥ 0.4 ng/ml and normal IGF-1), respectively. Fg-SRL treatment after surgery were provided for 21 patients including 12 patients with fg-SRL before surgery. Therefore, we analyzed the preoperative fg-SRL data in 12 patients with both preoperative and postoperative fg-SRL treatment. Finally, we investigated the difference of response, modality, and duration of fg-SRL therapy between the LHRH responders and nonresponders, in 43 cases with fg-SRL (without pituitary surgery: 3 cases, preoperative: 31 cases, postoperative: 9 cases). The modalities were daily octreotide 100 μg, or octreotide long-acting release (LAR) 10–30 mg or lanreotide 60–120 mg, every 4 weeks (Table 1). The response to fg-SRL therapy was evaluated and expressed as a percentage as follows: (nadir IGF-1 level − basal IGF-1 level)/basal IGF-1 level. We investigated the fg-SRL efficacy in short term because the limited subjects in our cohort were not enough to evaluate that in long term. Therefore, nadir IGF-1 level was defined as the minimum level 1–3 months (median month: 3) after initiation of fg-SRL therapy. Additionally, we compared the difference of fg-SRL efficacy between LHRH responders and nonresponders in only OGTT paradoxical nonresponders for excluding the influence of overlap with OGTT paradoxical responders with ≥ 20% increased response to OGTT.

**Table 1:** Treatment modality and duration in the evaluation of fg-SRL efficacy of LHRH nonresponders and responders

	LHRH nonresponders	LHRH responders	*P* value
Patients, n	33	10	
^a^Modality of fg-SRL therapy, n (%)			0.34
100 μg of octreotide	0 (0)	1 (10)	
Octreotide LAR 20 mg	20 (61)	4 (40)	
Lanreotide LAR 60 mg	1 (3)	0 (0)	
Lanreotide LAR 90 mg	11 (33)	5 (50)	
Lanreotide LAR 120 mg	1 (3)	0 (0)	
^b^Duration of all fg-SRL therapy, m	3 (1–3)	3 (1.75–3)	0.56
^b^Duration of each fg-SRL therapy modality, m			
100 μg of octreotide		1	
Octreotide LAR 20 mg	2 (1–3)	3 (1.5–3)	0.34
Lanreotide LAR 60 mg	3		
Lanreotide LAR 90 mg	3 (2.75–3)	3 (2.5–3)	1.00
Lanreotide LAR 120 mg	3		

^a^The number of patients with fg-SRL therapy, number (n). Data are presented as n (%)

^b^Fg-SRL therapy duration, months (m). Data are presented as the median (IQR)

### Immunohistochemical evaluation

**Fig. 2:**
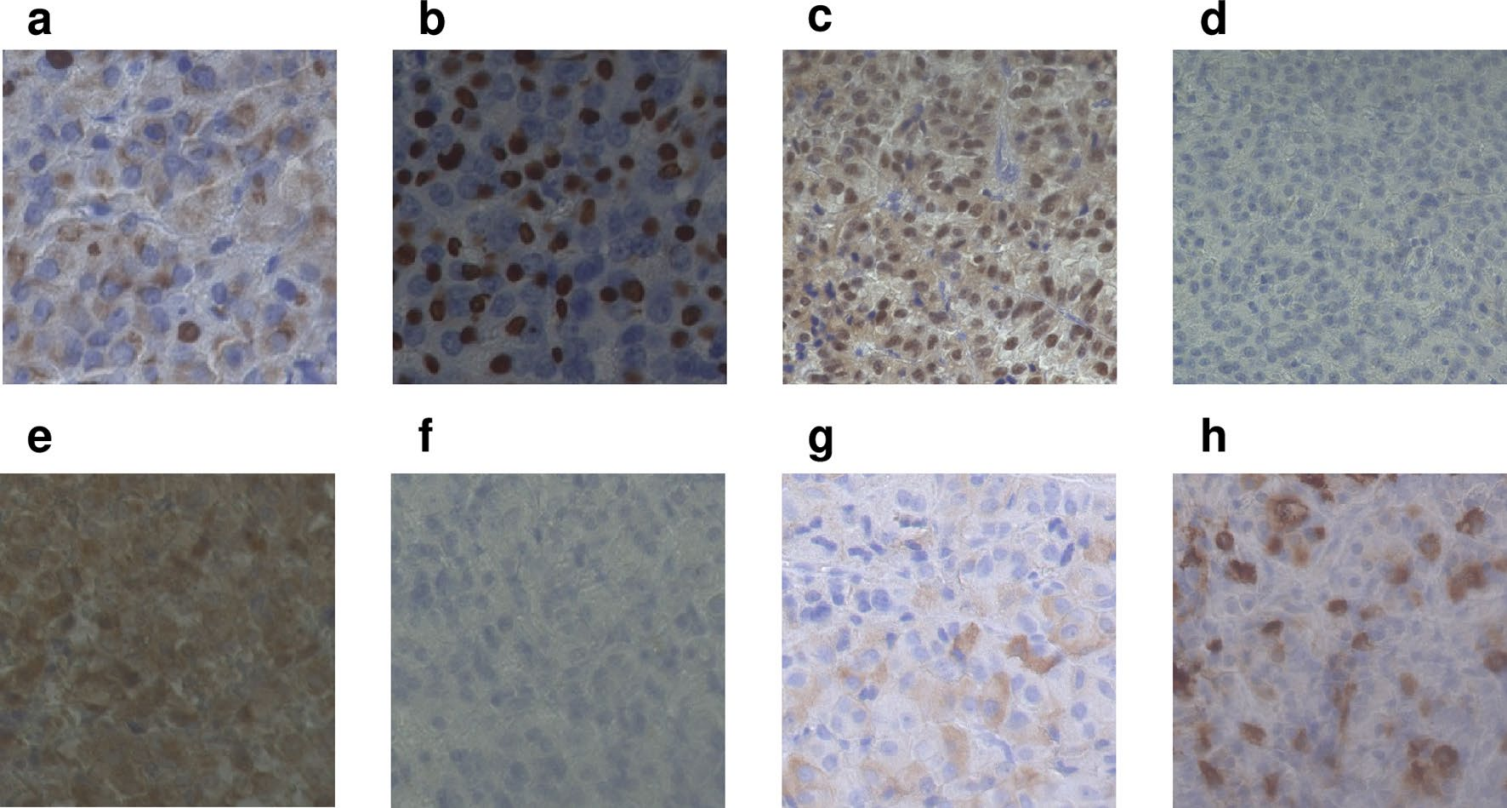
Representative immunohistochemistry in pituitary tumors of acromegaly. **a** Densely granulated type, **b** Sparsely granulated type, **c** Positive for SF-1, **d** Negative for SF-1, **e** Positive for GnRHR, **f** Negative for GnRHR, **g** LH and **h** FSH

Pathological evaluation was performed in 105 patients who underwent surgery at our hospital, and tumor tissues were obtained, including 31 patients investigated after preoperative fg-SRL treatment. Immunohistochemical studies were performed using the DAKO Autostainer Link 48 system (EnVision FLEX Mini kit, High pH, K8023; Dako A/S, Glostrup, Denmark). The sections were incubated with anti-SF-1 (Perseus Proteomics Cat# PP-N1655-0C, RRID:AB 2904221), anti-GnRHR (Abcam Cat# ab183079, RRID:AB 3083538), anti-LH (Agilent Cat# M3502, RRID:AB 2135325), anti-FSH (Agilent Cat# M3504, RRID:AB 2079146), anti-cytokeratin (BD Biosciences Cat# 349205, RRID:AB 2134314), anti-SSTR2A (Abcam Cat# ab134152, RRID:AB 2737601), and anti-SSTR5A (Abcam Cat# ab109495, RRID:AB_10859946). The transcription factor SF-1 was considered as positive when the cell nuclei of some cells were stained (Fig. 2). Positive for GnRHR, LH and FSH expression was defined as stained cell cytoplasm (Fig. 2). We evaluated the cytoplasmic distribution of cytokeratin and somatostatin receptor (SSTR) 2 expression as fg-SRL efficacy factors [9]. The cytoplasmic distribution of cytokeratin was classified into the densely granulated (DG) type, sparsely granulated (SG) type, and intermediate (IM) type, a mixture of both DG and SG, by immunostaining with CAM5.2 [10] (Fig. 2). SSTR2 and SSTR5 expression was assessed using the immunoreactive score (IRS), as previously published [11, 12]. Briefly, the IRS (0–12) is the product of the percent immunoreactivity score (0, 0% stained cells; 1, < 10%; 2, 10–50%; 3, 51–80%; 4, > 80%) and the staining intensity score (0, absent; 1, weak; 2, moderate; 3, strong). The median SSTR2 IRS in the entire cohort was 10.5 (IQR 4.5 to 12). The median SSTR5 IRS in the entire cohort was 8.5 (IQR 0.75 to 12).

### Evaluation of pituitary tumors on magnetic resonance imaging (MRI)

Radiological data were recorded on MRI scans obtained at diagnosis in 112 untreated patients. We measured the maximum diameter of the pituitary tumor. The T2-weighted MRI signal intensity of the tumor was assessed as an fg-SRL efficacy factor [13]. According to a previous study, we defined T2-weighted hypointensity in the pituitary tumor as a signal intensity lower than that of the white matter [13]. T2-weighted hyperintensity in the tumor was defined a signal intensity greater than that of the gray matter. T2-weighted isointensity in the tumor was defined as a signal intensity that was both greater than that of the white matter and lower than that of the gray matter. In 2 postoperative cases, because of MRI evaluation at the previous hospital, we could not confirm their pituitary tumor image at diagnosis. Therefore, these two cases were excluded from the analysis of radiological findings.

### Statistical analysis

Data on the clinical features are presented as the median and IQR (first and third quartiles). Differences between groups were analyzed using the χ^2^ test and Wilcoxon test. Correlation analysis was evaluated by Pearson’s correlation coefficient. *P* = 0.05 was considered to denote statistical significance. JMP Pro software, version 17.1.0, for Windows (SAS Institute, Cary, NC) was used for all analyses.

## Results

### Clinical characteristics of LHRH responders and nonresponders

An increased GH response to LHRH loading was seen in 33 patients (28.9%), with a median increase of 71% (IQR 41 to 167%). The clinical characteristics of the LHRH responders (n = 33) and nonresponders (n = 81) before acromegaly treatment are shown in Table 2. The sex, age, body mass index, and disease duration of the LHRH responders were not different from those of the nonresponders. No difference between the two groups was found in terms of basal serum levels of GH, IGF-1, LH, and FSH. In addition, there was no difference in the IGF-1 SD score between the two groups. LHRH responders had a significantly greater proportion of OGTT paradoxical responders than LHRH nonresponders [20% GH increase after OG loading: LHRH responders, 19 of 31 (61%); LHRH nonresponders, 28 of 80 (35%); *P* < 0.05, 30% GH increase after OG loading: LHRH responders, 18 of 31 (58%); LHRH nonresponders, 18 of 80 (23%); *P* < 0.01; Table 2]. The GH increase rate observed during OGTT exhibited a significant correlation with that of LHRH loading (*ρ* = 0.25; *P* < 0.01). There was no difference in the proportion of TRH responders between the two groups.

**Table 2:** Clinical characteristics of LHRH nonresponders and responders

	LHRH nonresponders	LHRH responders	*P* value
Patients, n	81	33	
Sex			
Female/male (%)	39 (48)/42 (52)	21 (64)/12 (36)	0.15
Age, year	52 (40–60)	55 (49–61)	0.11
Body mass index, kg/m^2^	24.3 (22.1–26.8)	23.9 (22.3–26.9)	0.67
Disease duration, year	9 (5–17)	10 (3–16)	0.70
Endocrinological data			
GH, ng/ml	8.7 (3.5–27.1)	8.8 (4.2–16.3)	0.77
IGF-I, ng/ml	695 (438–1006)	559 (439–938)	0.44
IGF-1 SD score	7.5 (5.2–9.5)	6.7 (5.3–9.4)	0.58
LH, mIU/ml	4.2 (2.0–17.2)	11.5 (1.7–18.1)	0.57
FSH, mIU/ml	12.9 (6.6–56.5)	34.5 (6.4–62.2)	0.66
Increased Response of GH to LHRH loading, %	10 (0–17)	71 (41–167)	< 0.01
OGTT paradoxical (20% increase) nonresponders/responders (%)	52 (65)/28 (35)	12 (39)/19 (61)	< 0.05
OGTT paradoxical (30% increase) nonresponders/responders (%)	62 (78)/18 (23)	13 (42)/18 (58)	< 0.01
TRH nonresponders/responders (%)	22 (28)/58 (73)	6 (18)/27 (82)	0.30
Histopathological findings			
Patients, n	73	32	
Densely/intermediate/sparsely granulated type (%)	50 (68)/10 (14)/13 (18)	29 (91)/2 (6)/1 (3)	< 0.05
Radiological findings			
Patients, n	80	32	
Tumor diameter, mm	14.9 (10.2–23.0)	13.9 (11.3–19.4)	0.64
MRI signal intensity			
Hypo/iso/hyper intensity (%)	24 (30)/22 (27)/34 (43)	18 (56)/8 (25)/6 (19)	< 0.05

Data are presented as the median (IQR) or n (%)

### Pathological findings

**Fig. 3:**
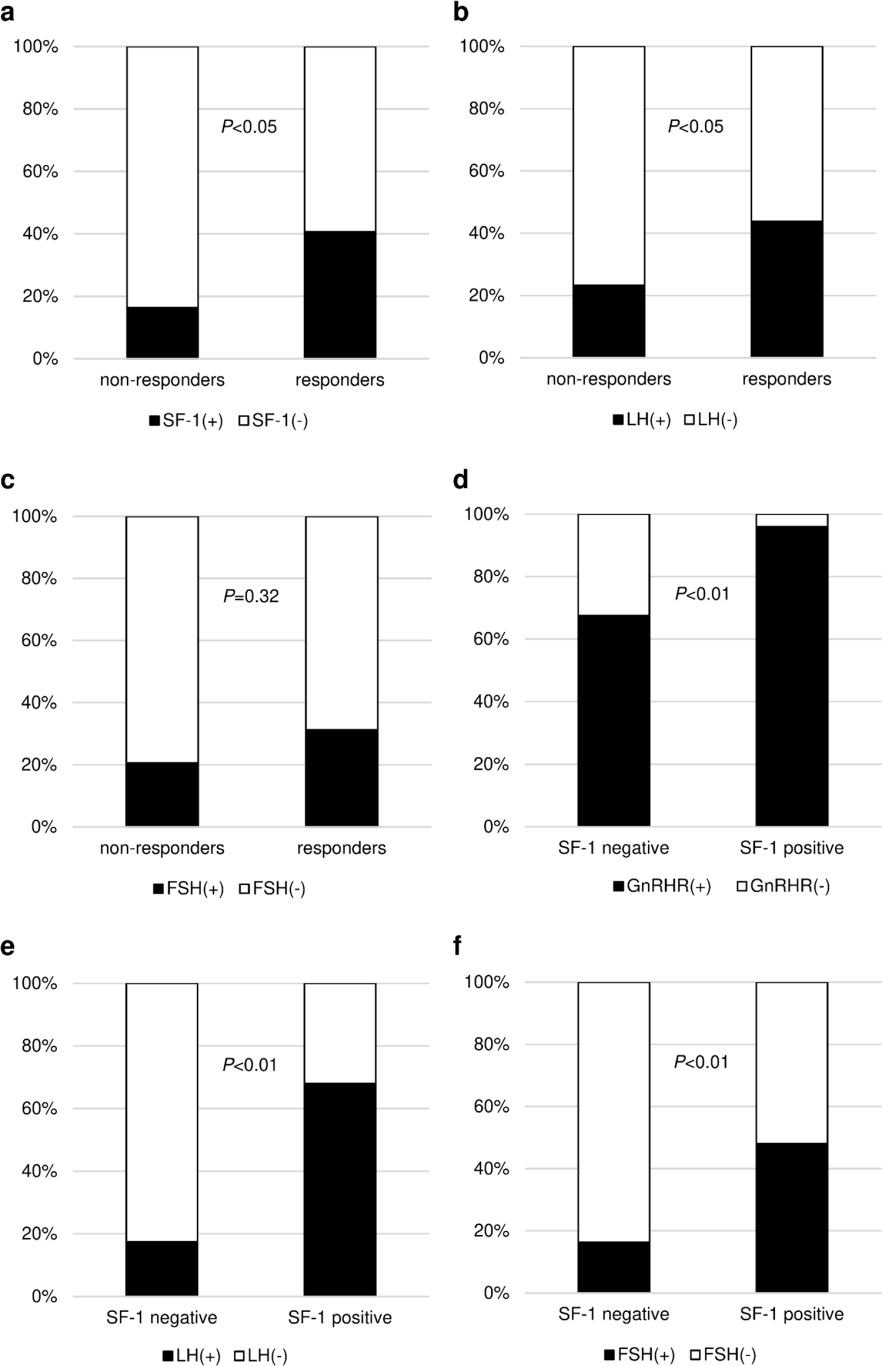
Histopathological classification by **a** SF-1, **b** LH, and **c** FSH expression, in LHRH nonresponders and responders. Histopathological classification by **d** GnRHR, **e** LH, and **f** FSH expression, in SF-1 negative and positive tumors

**Fig. 4:**
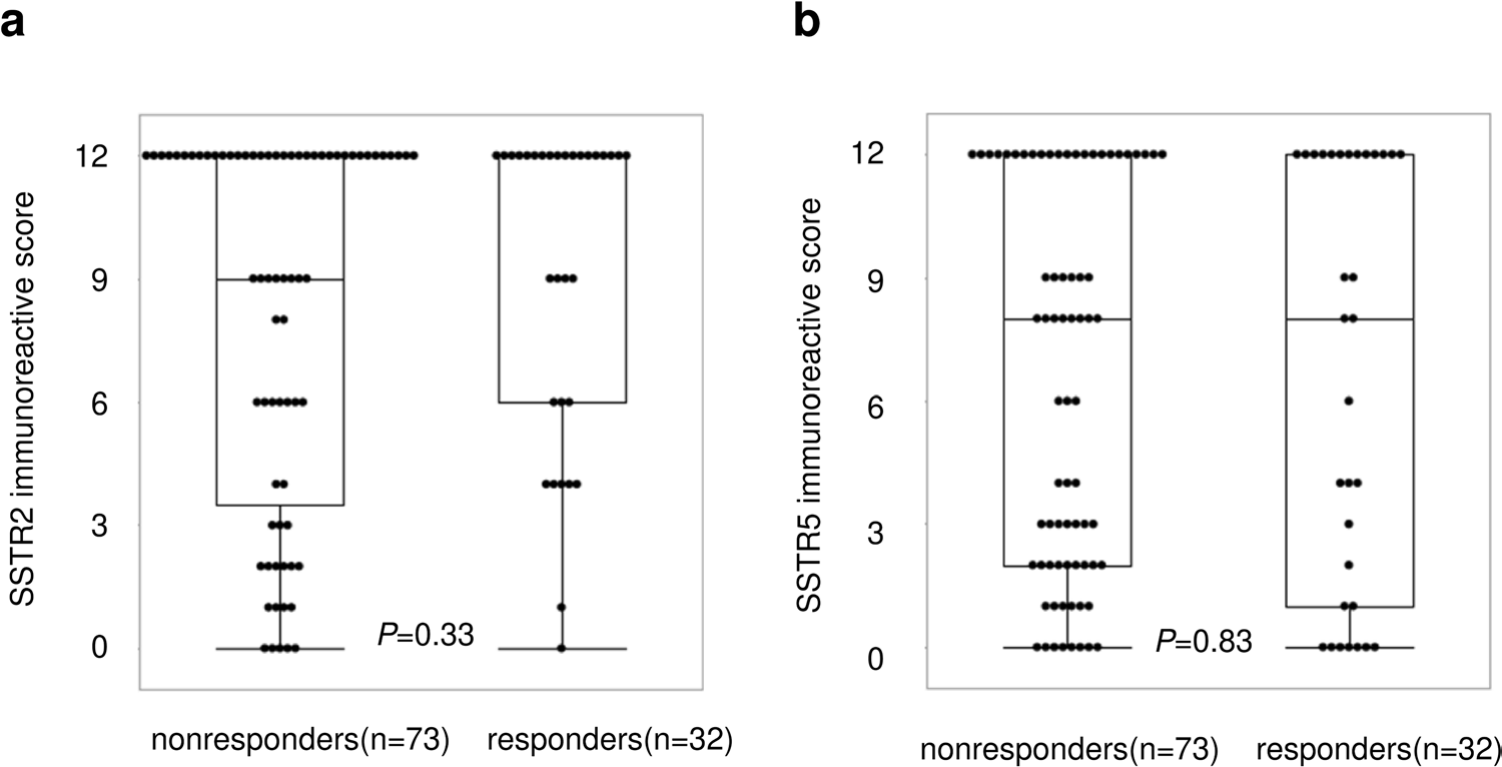
**a** SSTR2 immunoreactive score in LHRH nonresponders and responders. Data are presented for individual patients as a box plot. **b** SSTR5 immunoreactive score in LHRH nonresponders and responders. Data are presented for individual patients as a box plot

SF-1, GnRHR, LH, and FSH were positive in various proportions of cells within the tumor, whereas GH was positive in all cells throughout the tumor (Data not shown). A high proportion of SF-1 and LH positivity was found in the LHRH responders than in the nonresponders [SF-1: 13 of 32 (41%) vs. 12 of 73 (16%); P < 0.05, LH: 14 of 32 (44%) vs. 17 of 73 (23%); *P* < 0.05, Fig. 3]. FSH expression in the LHRH responders and nonresponders was 10 of 32 (31%) and 15 of 73 (21%), respectively (*P* = 0.32, Fig. 3). In addition, the proportion of GnRHR, LH and FSH positivity were higher in pituitary tumor with SF-1 expression than that without the expression [GnRHR: 24 of 25 (96%) vs. 54 of 80 (68%); *P* < 0.01, LH: 17 of 25 (68%) vs. 14 of 80 (18%); *P* < 0.01, FSH: 12 of 25 (48%) vs. 13 of 80 (16%); *P* < 0.01, Fig. 3]. The proportion of LHRH responders was higher in pituitary tumor with SF-1 expression than that without the expression [13 of 25 (52%) vs. 19 of 80 (24%); *P* < 0.05]. In addition, a significantly greater increased rate of GH after LHRH loading was observed in pituitary tumor with SF-1 expression than that without the expression [31.7% (IQR 14.0% to 207%) vs. 14.0% (IQR 2.35% to 27.9%), *P* < 0.01]. No difference was found in the increased rate of GH after LHRH loading between pituitary tumor with and without GnRHR expression [14.0% (IQR 4.34% to 23.1%) vs. 21.0% (IQR 5.62% to 47.7%), *P* = 0.11]. A high proportion of somatotroph tumors with a DG type was found in the LHRH responders than in the nonresponders [29 of 32 (91%) vs. 50 of 73 (68%); *P* < 0.05; Table 2]. While no difference was found in the SSTR2 IRS between the LHRH responders and nonresponders [12 (IQR 6 to 12) vs. 9 (IQR 3.5 to 12), *P* = 0.33, Fig. 4], the SSTR2 IRS was positively correlated with the decrease in GH after octreotide administration (*ρ* = − 0.46; *P* < 0.01). No difference was found in the SSTR5 IRS between the LHRH responders and nonresponders [8 (IQR 1 to 12) vs. 8 (IQR 2 to 12), *P* = 0.83, Fig. 4].

### Radiological findings in LHRH responders and nonresponders

No differences were found in the maximum diameter of the pituitary tumor between LHRH responders and nonresponders [tumor diameter, 13.9 mm (IQR 11.3 to 19.4) vs. 14.9 mm (IQR 10.2 to 23.0), *P* = 0.64; Table 2]. In contrast, a significantly greater proportion of pituitary tumors with T2-weighted hypointensity was observed for the LHRH responders than for the nonresponders (*P* < 0.05; Table 2).

### Response to bromocriptine, octreotide and fg-SRL therapy in LHRH responders and nonresponders

**Fig. 5:**
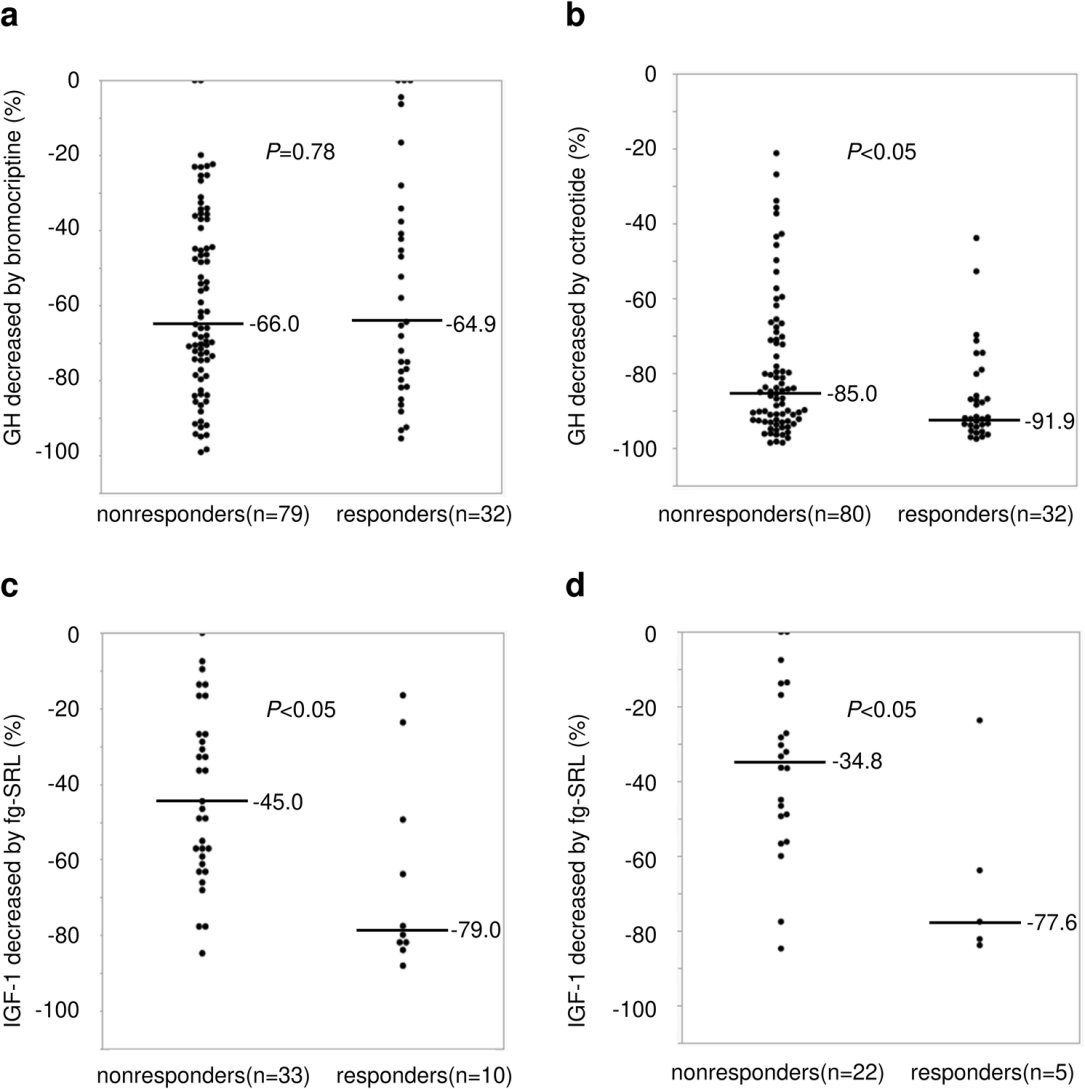
Percentage of GH decrease during **a** bromocriptine test and **b** octreotide test in LHRH nonresponders and responders. Percentage of IGF-1 decrease after fg-SRL treatment **c** in LHRH nonresponders and responders and **d** in LHRH nonresponders and responders without OGTT paradoxical responses (20% GH increase). Data are presented for individual patients with median group values

No difference was found in the decrease in GH after bromocriptine tests [− 64.9% (IQR − 81.2% to − 35.1%) vs. − 66.0% (IQR − 78.6% to − 39.4%), *P* = 0.78, Fig. 5]. A significantly greater suppression rate of GH was observed in the LHRH responders than in the nonresponders in octreotide tests [− 91.9% (IQR − 94.2% to − 81.6%) vs. − 85.0% (IQR − 92.6% to − 70.4%), *P* < 0.05, Fig. 5]. No difference was found in modality and duration of fg-SRL treatment [modality: *P* = 0.34, 3 months (IQR 1 to 3) vs. 3 months (IQR 1.75 to 3), *P* = 0.56, Table 1]. After fg-SRL therapy, the decrease in IGF-1 levels in LHRH responders was also greater than that in nonresponders [− 79.0% (IQR − 82.7% to − 43.0%) vs. − 45.0% (IQR − 60.7% to − 22.0%), *P* < 0.05, Fig. 5]. Moreover, in the analysis of only OGTT paradoxical nonresponders, the LHRH responders had a greater IGF-1 reduction by fg-SRL than the LHRH nonresponders (− 77.6% (IQR − 83.0% to − 43.8%) vs. − 34.8% (IQR − 51.1% to − 16.1%), *P* < 0.05, Fig. 5).

## Discussion

We observed a certain proportion of LHRH responders in acromegaly patients similar to that of prior studies [5, 14, 15]. We demonstrated that the LHRH responders had a higher frequency of pituitary tumors positive for SF-1, GnRHR, and LH. Similarly, a significantly greater increased rate of GH after LHRH loading was found in pituitary tumor with SF-1 expression than that without the expression. Therefore, the increased GH response to LHRH loading reflected the pathophysiological characteristics of acromegaly. Additionally, LHRH responders had a greater reduction in IGF-1 by fg-SRL treatment than LHRH nonresponders. Furthermore, GH reduction in the octreotide test was greater in LHRH responders. The proportion of LHRH responders with fg-SRL efficacy factors, such as T2-weighted hypointensity and a DG type, was significantly higher than that of LHRH nonresponders. Therefore, the increased GH response to LHRH might reflect fg-SRL efficacy. Regarding the GH response to tests other than LHRH, the LHRH responders demonstrated a higher proportion of OGTT paradoxical responders than LHRH nonresponders.

According to the 2022 WHO classification of pituitary tumors, somatotroph tumors and gonadotroph tumors express PIT-1 and SF-1, respectively. On the other hand, plurihormonal tumors express multiple combinations of pituitary transcription factors, including PIT-1 and SF-1 [6]. Therefore, pituitary tumor of LHRH responders included various proportions of plurihormonal pituitary tumor. Rymuza et al. reported that approximately 20% of somatotroph tumors expressed SF-1 [16]. It was shown that multiple synchronous pituitary neuroendocrine tumors were more often detected than previously believed [17]. Fookeerah et al. reported that some pituitary tumors expressing PIT-1 and SF-1 were positive for gonadotropin [18]. Matthias et al. reported a notable prevalence of PIT-1 and SF-1 co-expression in densely granulated somatotroph tumors, exhibiting distinctive molecular, histopathological, and clinical features [19]. Acromegaly with prolactin-cosecreting pituitary tumors has been found to have a mammotroph-like phenotype, such as a paradoxical GH response to TRH loading and dopamine agonist effectiveness [20]. Therefore, LHRH responders might have a gonadotroph-like phenotype. In addition, a high proportion of GnRHR expressing pituitary tumor was found in LHRH responders than in LHRH nonresponders. Therefore, the increased GH response to LHRH stimulation in acromegaly could partly depend on GnRHR. However, in addition to GnRHR expression, other mechanisms such as intracellular signaling might be required for the response because of no difference in the response rate between pituitary tumor with and without GnRHR expression. On the other hand, no significant difference in serum LH and FSH levels was observed between the LHRH responders and nonresponders. Although gonadotroph tumors express SF-1, most of them do not oversecrete gonadotropin. Therefore, serum gonadotropin levels might not be different between LHRH responders and nonresponders.

Older age, smaller tumor size, lower signal intensity on T2-weighted MRI, greater response to octreotide loading, DG type, mutations in the stimulatory G-protein α subunit, lower Ki-67 index, higher SSTR2 expression, no aryl hydrocarbon receptor interacting protein mutation, and paradoxical GH response to OGTT were considered fg-SRL efficacy factors for acromegaly [3, 4, 9]. In addition, the increased GH response to LHRH might reflect fg-SRL efficacy. On the other hand, there was no significant difference in SSTR2 IRS between LHRH responders and nonresponders although LHRH responders had some parameters of fg-SRL efficacy. However, the SSTR2 IRS was positively correlated with the decrease in GH by octreotide in the present study similarly to the previous study [12]. Therefore, the lack of a difference in SSTR2 IRS might be attributed to the limited number of participants. However, fg-SRL efficacy in LHRH responders regardless of SSTR2 IRS might represent a difference in intracellular signal transduction of somatotroph tumors between LHRH responders and nonresponders. Additionally, OGTT paradoxical response, more frequent in LHRH responders, was reported to have relationship with the fg-SRL efficacy [1, 3, 4]. Therefore, LHRH responders suggests fg-SRL efficacy.

As there was no difference in SSTR5 IRS between LHRH responders and nonresponders, it was suggested that the increased GH response to LHRH was not related to the efficacy of the SSTR5 ligand. However, because there were few patients who underwent treatment with an SSTR5 ligand, such as pasireotide, we could not evaluate whether the efficacy of the SSTR5 ligand was different between LHRH responders and nonresponders. Therefore, the relationship between the increased GH response to LHRH and SSTR5 ligand efficacy is still unclear.

In the present study, there was no difference in GH reduction by bromocriptine between LHRH responders and nonresponders. It has been reported that bromocriptine was more effective in acromegaly patients with an increased GH response to TRH loading alone than in those with an increased GH response to both TRH and LHRH loading [5]. Therefore, there are some characteristic differences between LHRH and TRH responders.

The primary limitation of this study is that it is retrospective. Prospective research is needed. Second, only the short-term efficacy of fg-SRL was evaluated. Moreover, fg-SRL treatment period and dosage was not uniformed although there was no significant difference of fg-SRL treatment period between LHRH responders and nonresponders. Third, the number of patients was limited, although baseline clinical characteristics such as sex, age, and body mass index were not different. A larger prospective long-term study should reconfirm the clinical and pathological characteristics of LHRH responders.

In conclusion, the present study demonstrated that the increased GH response to LHRH was related to the gonadotroph-related characteristics. This response may evaluate the biological characteristics of somatotroph tumors noninvasively.

## Data Availability

Some or all datasets generated and/or analyzed during the current study are not publicly available but are available from the corresponding author on reasonable request.
